# Uncovering the dual roles of peripheral immune cells and their connections to brain cells in stroke and post-stroke stages through single-cell sequencing

**DOI:** 10.3389/fnins.2024.1443438

**Published:** 2024-11-20

**Authors:** Zheng Xu, Fan Yang, Lifang Zheng

**Affiliations:** ^1^Department of Neurology, Southern University of Sciences and Technology Yantian Hospital, Shenzhen, China; ^2^Institute of Biomedicine and Biotechnology, Shenzhen Institute of Advanced Technology, Chinese Academy of Sciences, Shenzhen, China

**Keywords:** stroke, post-stroke, scRNA seq, PBMC, dual roles

## Abstract

Ischemic stroke is a cerebrovascular disease that affects the blood vessels and the blood supply to the brain, making it the second leading cause of death worldwide. Studies suggest that immune cells play a dual role during the inflammatory and recovery phases of stroke. However, in-depth investigations of specific cell subtypes and their differentiation trajectories remain to be elucidated. In this review, we highlight the application of single-cell RNA sequencing (scRNA-seq) for the unbiased identification of cell heterogeneity in brain and peripheral blood mononuclear cells (PBMCs) during and after a stroke. Our goal is to explore the phenotypic landscape of cells with different roles in this context. Specifically, we provide an overview of the roles, cell surface markers, immune cell-released cytokines, and intercellular interactions identified in major immune cells during and after stroke, as identified by different technologies. Additionally, we summarize the connection between immune cells in peripheral blood and the brain via their differentiation trajectories. By synthesizing the application of scRNA-seq in the combined analysis of PBMCs and brain tissue at higher sampling frequencies, we aim to unveil the dual role of peripheral immune cells, which could facilitate the development of new treatment strategies for ischemic stroke.

## 1 Introduction

Stroke occurs when the supply of oxygen and blood to the brain is reduced or interrupted. There are two main types of stroke related to blood deficiency: ischemic stroke (IS) and hemorrhagic stroke (HS). Approximately 75 to 80% of clinical stroke cases are acute ischemic stroke (AIS), which is the second leading cause of death globally (World Health Organization, 2020). Reperfusion therapy during the acute phase is one of the most effective treatments for AIS patients. However, even with reperfusion therapy, more than 40% of ischemic stroke patients experience the secondary complication, such as post-stroke infections and post-stroke dementia, which is due to stroke-induced immunosuppression (Rocco et al., 2013).

Immune cells play a dual role during the inflammatory and recovery phases of stroke. These cells actively participate in the inflammatory response triggered by ischemic injury, which can contribute to secondary damage and exacerbate neurological deficits. Conversely, these immune cells are also involved in the reparative processes during the subacute and chronic stages, promoting tissue repair and functional recovery (Ma et al., 2021). Therefore, early detection of specific cellular subpopulations or unique cellular markers provides valuable insights for timely interventions against stroke progression and enhances predictive capabilities for stroke outcomes.

A growing body of research shows a complex interaction between the central nervous system (CNS) and the peripheral immune system triggered by AIS (Ma et al., 2021; Zhang S. R. et al., 2021; Greenhalgh et al., 2020). The destruction of neural cells results in the release of damage-associated molecular patterns (DAMPs) into the extracellular environment, stimulating post-stroke inflammation by promoting the expression of pro-inflammatory cytokines, chemokines, and adhesion molecules. This, in turn, facilitates leukocyte infiltration into the brain (Salvador and Kipnis, 2022). This inflammatory response disrupts the blood–brain barrier (BBB), regulating its permeability and exposing neuronal antigens to the periphery, further stimulating inflammation (Huang et al., 2021). Consequently, post-stroke immunological changes extend beyond the brain, affecting other peripheral organs, such as the blood, spleen, and gut (Pennypacker, 2014).

Among these, peripheral blood immune cells respond to cerebrovascular injury by altering their counts and activities, making these dynamics potential targets for stroke diagnosis and understanding stroke etiology (Qiu et al., 2021). For example, neutrophils, the most abundant type of white blood cells, often increase in number immediately after a stroke. Their precursors, the neutrophil-to-lymphocyte ratio (NLR), have been studied as a prognostic marker for stroke severity and outcomes (Liu et al., 2020).

Natural killer (NK) cells have been tested as another biomarker; an elevated count of circulating NK cells, coupled with reduced expression levels of interferon-gamma (IFN-*γ*) or perforin by these cells, has been proposed as an indicator of increased risk for post-stroke infection (Chen et al., 2019). These immune responses correlate with the severity and outcomes of stroke, making NK cells promising biomarkers for stroke prediction.

Nevertheless, a comprehensive understanding of peripheral blood immune cells and their dynamics after stroke is essential for harnessing the post-stroke immune response and improving post-stroke outcomes (Wardlaw et al., 2022). However, current analyses often rely on bulk measurements of PBMCs, which average signals from thousands of cells.

This averaging makes it challenging to detect subtle changes in the disease process, as it masks the diversity and unique characteristics of individual cells. Furthermore, high-throughput technologies such as genomics, transcriptomics, and metabolomics, useful for identifying biomarkers for diagnosis, lack the precision required to distinguish the dual functional states and transcriptional profiles of individual immune cell subsets during AIS. This limitation leads to an incomplete understanding of their differentiation in relation to stroke pathogenesis and recovery (Montaner et al., 2020; Harpaz et al., 2017).

**Figure 1:**
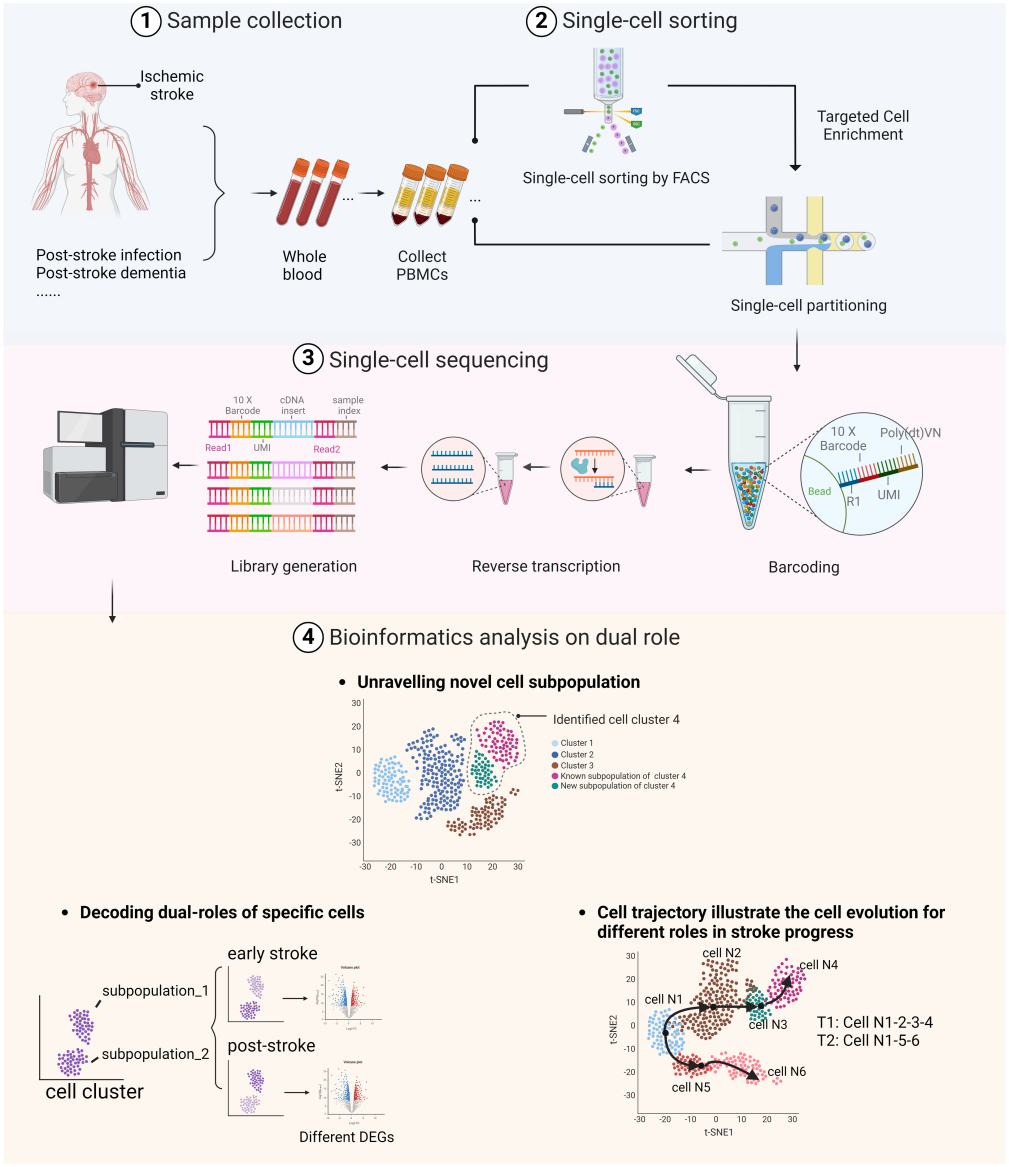
Dual roles of peripheral immune cells in stroke and post-stroke stages as identified through scRNA-seq. (1) Whole blood samples were collected from stroke patients, including those who have post-stroke comorbidities. (2) After extracting the peripheral blood mononuclear cells (PBMC), samples were sorted using fluorescence-activated cell sorting (FACS) technologies or directly processed through single-cell partitioning. (3) The library was prepared through barcoding and reverse transcription to complementary DNA (cDNA), followed by sequencing of the treated DNA using the selected sequencing platform. (4) Bioinformatics analyses illustrate the dual roles of immune cells in AIS by identifying specific cell types and subtypes, uncovering various cell states and activation levels, discovering rare or novel cell subtypes, and elucidating differentiation trajectories and transition states. Image created with Biorender.com, with permission.

Single-cell RNA sequencing (scRNA-seq) technologies push the boundaries of high-throughput studies by enabling the creation of a high-resolution reference map of cell transcriptional states. scRNA-seq facilitates the examination of individual cells within heterogeneous populations in stroke conditions (Zheng et al., 2022; Guo et al., 2021). Its benefits include the following:

- *Cellular heterogeneity*: scRNA-seq provides detailed insights into the heterogeneity of cell populations by identifying distinct cell types and states within a mixed population.
- *Gene expression dynamics*: This technology captures the dynamic range of gene expression in individual cells by offering a snapshot of transcriptional activity at a given time.
- *Developmental trajectories*: scRNA-seq can trace the developmental pathways of cells by mapping their differentiation paths and identifying key regulatory genes at various stages.
- *Cell-specific responses*: It allows for the investigation of how individual cells respond to environmental changes or treatments by revealing cell-specific regulatory mechanisms (Figure 1).

Given that access to brain tissue from patients is limited, most recent research on stroke using scRNA-seq relies on mouse brain tissue (Table 1). However, one study found that only 9% of identified pathways were shared between human and mouse stroke samples during the acute phase, while 47% were shared at the subacute phase. This disparity complicates the translation of findings from mouse studies to human clinical settings (Garcia-Bonilla et al., 2024). Therefore, combining the cellular responses observed in the peripheral blood of stroke patients with studies conducted on mouse models is essential to fully interpret the results. This highlights the need for a comprehensive understanding of the dual role of peripheral blood cells in stroke.

**Table 1:** Bulk-RNA-seq, scRNA-seq, and spatial transcriptomics studies reveal cellular and molecular heterogeneity in IS.

Study	Sample source	Platform	Method	Time point	Highlighted markers or pathways
Guo et al. (2021)	Dissociated brain tissue of mice	10× Genomics & Illumina Novaseq6000	scRNA-seq & BulkRNA-seq	24 h after IS	*Gadd45b* in microglia, *Cyr61* in astrocytes, and *Sgk3* in oligodendrocytes
Zheng et al. (2022)	Dissociated brain tissue of mice	10× Genomics	scRNA-seq	24 h after IS	17 principal brain clusters with cell-type specific gene expression patterns; Markers in Table 5
Li et al. (2022)	Dissociated brain tissue of aged mice	10× Genomics	scRNA-seq	Day 3	*Il1b, Mmp9*, and *Apoe* for stroke pathogenesis*; Aif1 (Iba1)* microglial; *Sall1* microglia and *Cd44* in infiltrated myeloid cells*; CD11c (Itgax)* in DC, NKs, and monocytes/macrophages*; Flt3* in DC for aged stroke brain
Beuker et al. (2022)	Dissociated brain tissue of mice; blood from mice tail vein; Middle cerebral artery of human brain	10× Genomics	scRNA-seq	24 h and 72 h after IS in mice; Median 3 days after stroke in human	*Spp1, Fabp5, Gpnmb, Ctsb, Ctsl, Lgals3, Lpl, Fth1, Cd63*, and *Ctsd* in stroke-associated myeloid cells
Cho et al. (2022)	Peripheral blood of human	Illumina Bio-Rad SureCell	scRNA-seq	24 h after IS	Dendritic cell-related CD14+ monocytes and NK cell-related CD14+ monocytes; EIF2 signalling pathway
Garcia-Bonilla et al. (2024)	Peripheral blood& Brain tissue of mice	Drop-seq, Bio-rad	scRNA-seq	Day 0, Day 2, Day 14	Neutrophils; comparison of cell origin (brain resident vs. recruited cells), cellular localization (periphery vs. brain) time post stroke (acute vs. subacute) and age (young vs. aged mice)
Han et al. (2024)	Dissociated brain tissue of mice	10× Genomics & 10× Genomics	Spatial RNA-seq & scRNA-seq	Spatial RNA-seq: 18 min for Sham or 24 min for PT; scRNAseq: Day 3	*Lgals9* in long-term functional recovery; LGALS9-CD44 signaling pathway
Jin et al. (2023)	Dissociated brain tissue of adult and aged mice	10X Genomics	scRNA-seq	Acute (Day 3) and chronic (Day 14)	the age-related decay mechanisms in brain repair; MG/MΦ as effective targets for promoting stroke recovery
Simats et al. (2024)	peripheral organs (heart, lung, liver, spleen and/or blood) and bone marrow	10X Genomics	scRNA-seq; BulkRNA-seq; snATAC-seq; BulkATAC-seq;	Patients:3 and 6 months Mice:1, 3 and 6 months	Acute brain ischemia leads to persistent innate immune memory; IL-1b induces post-stroke-trained immunity
Gu et al. (2024)	Peripheral blood of mice	10× Genomics	scRNA-seq	Day 5 and Day 14	*C1q*, *Apoe*, *Hexb*, and *Fcer1g* of T cells promoting stroke recovery

To date, numerous studies have provided comprehensive reviews on the role of resident immune cells in the brain concerning ischemic stroke and their functions in regulating the acute phase of injury (Ma et al., 2021; Garcia-Bonilla et al., 2024; Qiu et al., 2022). However, studies using scRNA-seq to target immune cells in peripheral blood are rare (Cho et al., 2022), which limits the comprehensive understanding of the dual roles of PBMCs and their differentiation trajectories in the event of AIS.

In this review, we summarize the roles and fluctuations of peripheral immune cell populations and the differentiation trajectories of distinct cell types in the brain during the ischemic stroke and post-stroke stages using scRNA-seq. We aim to provide a synthesis of potential biomarkers within various cell subpopulations. Additionally, we discuss the advantages and potential limitations of scRNA-seq for future research.

## 2 Myeloid cells in peripheral blood mononuclear cells (PBMC)

Myeloid cells, derived from hematopoietic stem cells in the bone marrow, play critical roles during and after ischemic stroke. Monocytes and dendritic cells can be found in PBMC, while neutrophils are rarely detected due to higher cell density. The balance and timing of these myeloid cell responses are vital. Early aggressive inflammation can worsen outcomes, whereas later inflammation resolution and tissue repair support can enhance recovery (Letko Khait et al., 2021).

### 2.1 Monocytes

#### 2.1.1 Role of monocytes in IS

Monocytes are immune effector cells that normally circulate in the bone marrow, blood, and spleen without proliferation (Auffray et al., 2009). Monocytes migrate from peripheral blood circulation to the ischemic area after acute cerebral ischemia, accumulating in the ischemic region before differentiating into macrophages (Rajan et al., 2019). The role of monocytes/macrophages (MMs) in the ischemic brain tissue is still debatable. While some studies suggest they may have a dual role, their influence as protective or detrimental agents in this context has yet to be unequivocally determined (Wattananit et al., 2016; Werner et al., 2020). Thus, the variations in monocyte subsets identified in the PBMCs of stroke patients may reflect the diverse inflammatory profiles of these patients.

#### 2.1.2 Heterogeneity of monocytes in IS

Using flow cytometry, CD163^+^ monocytes are found to be associated with the severity of the IS using flow cytometry (Greco et al., 2021b). Human leucocyte antigen D-related (HLA-DR), CD64, and CD14 expression alterations are found in monocyte subsets, specifically with downregulation of HLA-DR expression on intermediate monocytes (Krishnan et al., 2021). This study also implemented flow cytometry to uncover the inversed association between stroke severity and CD69^+^CD4^+^ T cell phenotype (Krishnan et al., 2021). Furthermore, mass cytometry found IRF4, phosphorylation of key kinases (p)-*PLCg2* and *Arg-1* are significantly decreased in IS (Yao et al., 2021). Tie2-expressed monocytes (TEMs) have a higher level in IS patients present and are considered a diagnostic marker of neurodamage (Alrafiah, 2023). High expression levels of the cannabinoid-2 (CB2) receptor may be related to a protective response to limit damage in CD16^+^ circulating monocytes of IS patients (Greco et al., 2021a).

Traditionally, three main subsets of monocytes can be found in human PBMC based on CD14 & CD16 (Ziegler-Heitbrock et al., 2010), including (1) classical monocytes: CD14^++^CD16^−^, (2) intermediate monocytes: CD14^++^CD16^+^, and (3) non-classical monocytes: CD14^+^CD16^++^ (Table 2).

**Table 2:** Markers of human peripheral blood cell subsets by flow cytometry and scRNA-seq in IS.

Cell types	Subsets	Markers for flow cytometry	Marker genes for scRNA-seq	References
Neutrophils		SSC^hi^CD66b^+^CD16^+^	N/A	Krishnan et al. (2021)
Monocytes	Classical monocytes	HLA-DR^+^CD14^++^CD16^−^	S100A9, LYZ and CD14	Cho et al. (2022), Krishnan et al. (2021)
	Intermediate monocytes	HLA-DR^+^CD14^++^CD16^+^	FCGR3A	
	Non-classical monocytes	HLA-DR^+^CD14^+^CD16^++^	FCGR3A and MS4A7	
Dendritic cells	Conventional DC1	HLA-DR^+^CD14^−^CD16^−^CCR2^+^CD141^+^	XCR1, CLEC9A, BATF3	Cho et al. (2022), Garcia-Bonilla et al. (2024), Krishnan et al. (2021)
	Conventional DC2	HLA-DR^+^CD14^−^ CD16^−^CCR2^+^CD1c^+^	CD1c, CLEC10A, SIRPA (CD172a)	
	Plasmacytoid DC	HLA-DR^+^CD14^−^CD16^−^CCR2^+^CD123+	LILRA4, ITM2C, JCHAIN and PTPRS	
B cells	Plasmablasts	CD3ε^−^CD19^+^CD20^+^CD27^high^CD38^high^		Krishnan et al. (2021)
	Unswitched memory	CD3ε^−^CD19^+^CD20^+^CD27^+^IgD^+^		
	Switched memory	CD3ε^−^CD19^+^CD20^+^CD27^+^IgD^−^		
	Naïve	CD3ε^−^CD19^+^CD20^+^CD27^−^IgD^+^		
	Double-negative	CD3ε^−^CD19^+^CD20^+^CD27^−^IgD^−^		
T cells	γδ T cells	CD45^+^CD3ε^+^TCR-γδ^+^Vδ^+/−^	TRDC	Krishnan et al. (2021), Ge et al. (2024)
	CD4+ T cells	CD45^+^CD3ε^+^TCR-γδ^−^CD4^+^	CD3D, IL7R, CD3E, CD4	
	CD8+ T cells	CD45^+^CD3ε^+^TCR-γδ^−^CD8^+^	CD8A, CD3E	
	Central memory T (TCM) cells	CD45^+^CD3ε^+^CCR7^+^CD45RA^−^	S100A4, GPR183, CCR7, SELL	
	Naïve	CD45^+^CD3ε^+^CCR7^+^CD45RA^+^	CCR7, SELL	
	T cells re-expressing CD45RA (TEMRA)	CD45^+^CD3ε^+^CCR7^−^CD45RA^+^		

Additionally, a previous study revealed that intermediate and non-classical monocytes are developed from classical monocytes via transcriptomic analysis (Yona et al., 2013). Based on gene expression profiles by scRNA-seq targeting PBMC of IS patients, two distinct clusters of CD14^+^ monocytes were identified (Cho et al., 2022), including dendritic cell-related CD14^+^ monocytes and NK cell-related CD14^+^ monocytes (Table 3). The overall transcriptomic levels in monocytes from stroke patients indicate a shift toward inactivity, primarily due to the strong inhibition of EIF2 signaling. However, one specific subcluster displays a distinct profile of differentially expressed genes (DEGs) that are relevant to neurological disorders, suggesting that each cluster exhibits unique characteristics and activation timelines (Cho et al., 2022). These results underscore that the DEGs within this subcluster have the potential to be developed as prognostic or diagnostic biomarkers for ischemic stroke and its therapeutic interventions.

**Table 3:** Biomarkers and DEGs of human peripheral blood cells by scRNA-seq in IS.

Cell types	Subsets	Marker genes in scRNA-seq	Pathway	DEGs	References
Monocyte	Dendritic cell-related CD14+ monocytes	S100A9, LYZ, CD14, FCGR3A, PGD, CRIP1, XIST and TMEM176B	EIF2 signalling	Up: KLF10, NLRC5 and ISG15, HLA. DPA1, DPB1, DQB1, DRA and DRB1, interferon-related genes	Cho et al. (2022)
	NK cell-related CD14+ monocytes	S100A9, LYZ, CD14, CD8A, CD3D, IL7R	EIF2 signalling	Up:GZMA/B, PRF1, NKG7, IFITM1, KLRD1, GNLY, CDL5, CD247, MS4A1, HLA.DQA1	
NK cells		GNLY and NKG7	IFN-p38 MAPK pathway	Up: CX3CR1 and GIMAP7	Cho et al. (2022)
			Nuclear factor kappa B (NFκB) pathway	Down:H3F3B, EIF3H, RPL7, BTG1, CD74, HLA.DRB1, CD69, NFKBIA, TSC22D3, CD3D, TNFAIP3, DUSP1, LTB, ZFP36, IL7R, DUSP2, CXCR4	

### 2.2 Dendritic cells (DCs)

#### 2.2.1 Role of dendritic cells in IS

Dendritic cells (DCs) are highly effective antigen-presenting cells (APCs) that evolved from both myeloid cells and lymphocytes (Ludewig et al., 2016). DCs play a significant role in neurological disorders such as multiple sclerosis, Alzheimer’s disease, cerebral infarction (CI), and epilepsy (Sagar et al., 2017).

#### 2.2.2 Heterogeneity of dendritic cells in IS

DCs evolved from both myeloid cells and lymphocytes. Identifying DCs in blood samples can be tricky due to their rarity and the overlapping expression of markers with other immune cells. Currently, two main subsets of DCs were found: myeloid DCs and plasmacytoid DCs (pDCs) (Table 2). Common dendritic cell (DC) precursors in the bone marrow differentiate into plasmacytoid DCs (pDCs) or evolve into pre-DCs. These pre-DCs exit the bone marrow, circulate in the bloodstream, and ultimately mature into classical DCs (cDCs, also named myeloid DCs). pDCs and two types of cDCs are distinguished as CD1c^+^ and CD141^+^ DCs in humans. Studies reported reductions in total dendritic cells, cDCs, and pDCs in the peripheral blood of IS patients (Yilmaz et al., 2010). In patients with cerebrovascular stenosis, the proportion of cDCs in peripheral blood decreases as the severity of stenosis increases (Yilmaz et al., 2006).

Moreover, mild stroke patients show a slight proportional increase in peripheral cDCs (3.6% in control vs. 2.8% in mild ischaemic stroke) and pDCs (1% in control vs. 0.8% in mild ischaemic stroke) compared to the control group (Cho et al., 2022).

Typically, CCR2 is used as a marker to identify DCs in studies using flow cytometry (Krishnan et al., 2021). Based on the expression of CCR2, DCs can be further divided into functionally distinct populations: (1) conventional DC1: HLA-DR^+^CCR2^+^CD141^+^; (2) conventional DC2: HLA-DR^+^CCR2^+^CD1c^+^; and (3) plasmacytoid DC: HLA-DR^+^CCR2^+^CD123^+^ (Krishnan et al., 2021) (Table 2). A slight decrease of cDC1 in terms of frequencies is observed following a stroke, which is potentially attributable to their recruitment to the ischemic brain (Krishnan et al., 2021).

Conversely, a broader phenomenon of downregulated HLA-DR is observed, suggesting the pivotal roles of cDC2s and pDCs in priming T-cell responses and promoting type I interferon production in IS. In scRNA-seq, markers identifying cDCs include CD1c and CLEC10A, while typical markers identifying pDCs are LILRA4, ITM2C, JCHAIN, and PTPRS (Cho et al., 2022). Apart from the variations between these two subsets, the features of DCs are also expressed in CD14^+^ monocytes.

## 3 Stroke-related lymphocyte populations

Peripheral lymphocytes could reflect CNS inflammatory injury associated with AIS (Salvador and Kipnis, 2022). The initial neuroinflammatory response in the CNS after a stroke sends signals to the peripheral immune system, causing a post-stroke immunodepressive state in the ischemic brain (Meisel et al., 2005). The primary indicators of stroke-induced immunodepression (SIIS) include lymphopenia predominantly affecting CD4^+^ T cells, an increased neutrophil-to-lymphocyte ratio, and decreased expression of antigen-presenting molecules such as monocyte HLA-DR (Iadecola and Anrather, 2011; Chamorro et al., 2012).

### 3.1 T cells

#### 3.1.1 Role of T lymphocytes in IS

T lymphocytes (T cells) are crucial players in cellular adaptive immunity and serve important functions across numerous neurological disorders (Greenhalgh et al., 2020). There is an interplay among various T cell subtypes in the peripheral blood after ischemic stroke (Zhang D. et al., 2021). These T cell subtypes respond to signals from the injured brain and undergo changes that can either exacerbate or ameliorate stroke outcomes (Wang M. et al., 2023), each with its own unique functional roles. For example, Th1 and Th17 cells tend to promote pro-inflammatory responses, potentially worsening brain injury in stroke (Wang Y.-R. et al., 2023). Double-negative T cells (DNTs, CD3^+^CD4^−^CD8^−^ T-cells), which are found to exacerbate inflammation following a stroke, significantly increased in both the brain and peripheral blood of stroke patients after AIS. CD8^+^ T cells can cause neuronal cell death through direct cytotoxic effects or by producing pro-inflammatory cytokines, which leads to exacerbated tissue damage after a stroke (Zhang D. et al., 2021).

In contrast, regulatory T cells (Tregs) and natural killer T cells (NKT) can mitigate overactive inflammation. Tregs (CD4^+^CD25^+^) have a dual role in AIS, with increasing evidence supporting their predominantly protective functions. The proportion of Treg cells was notably increased after an AIS (Xiao et al., 2021), offering protective benefits by modulating the peripheral immune response at an early stage of post-AIS (within 24 h). NKT cells predominantly secrete Th2-type cytokines, including IL-10 and IL-5, acting in an immunosuppressive manner to mitigate the damage caused by inflammatory cells after AIS. Although NKT cells are not traditional T cells, their activation can lead to both pro-inflammatory and anti-inflammatory responses (Kumar and Delovitch, 2014).

Interestingly, *γ*δ-T cells (γδ-T) have pro-inflammatory properties but can also ameliorate stroke outcomes, including pathogen defense and the preservation of tissue equilibrium. Following the AIS, γδ-T cells are rapidly activated, initially playing a protective role against barrier infections by secreting cytokines such as IL-17 and IFN-γ (Balch et al., 2020; Alves de Lima et al., 2020). IL-17, primarily produced by γδ-T cells in the early stages of AIS, damages the blood–brain barrier, promotes immune cell infiltration, and contributes to neuronal apoptosis and autophagy (Alves de Lima et al., 2020; Wang et al., 2022). IL-17 is also linked to the development of post-stroke depression and other neuropsychiatric conditions, suggesting its accumulation after stroke could have broad impacts on patient recovery and mental health (Jian et al., 2019).

#### 3.1.2 Heterogeneity of T lymphocytes in IS

In flow cytometry, common cell markers used for T cells are CD45^+^CD3^+^. For γδ T cells, TCR^−^γδ^+^Vδ^+/−^ is a combination of cell markers, while CD4 and CD8 are used for CD4^+^ and CD8^+^ T cells. Among CD4 and CD8 T cells, effector memory T cells (TEM) and central memory T cells (TCM) can be found by CCR7^−^CD45RA^−^ and CCR7^+^CD45RA^−^, respectively (Table 2). Normally, CCR7 on naïve T cells is positive, and effector memory T cells can be identified with re-expressing CD45RA (TEMRA) (Krishnan et al., 2021). These results show decreased frequencies of TEMRA cells and find that CD69^+^CD4^+^ T cells are inversely correlated with stroke severity and are associated with naive and TCM cells.

In scRNA-seq, CD3D, CD3E, IL7r, and CD4 are reported to be used for CD4^+^ T cells and CD8A for CD8^+^ T cells (Table 2). CD4^+^ T cells are slightly increased in stroke patients by scRNA-seq, while CD8^+^ T cells are identical in control and stroke patients. However, the results from flow cytometry in this study show that CD4^+^ T cells and CD8^+^ T cells were decreased in patients who had a stroke, which did not align with the result in a study using scRNA-seq (Cho et al., 2022).

### 3.2 B lymphocytes

#### 3.2.1 Role of B lymphocytes in IS

B lymphocytes (B cells) are the key players in humoral immunity, critically involved in anti-microbe defense, antibody production, and cytokine secretion (Jain and Yong, 2022). However, how B cells modulate in the acute phase of ischemic stroke remains unclear. Some studies observed beneficial roles of B cells (Ren et al., 2011; Offner and Hurn, 2012), especially certain B cell subsets, which were found crucial to affect the different responses in a post-stroke stage. Regulatory B cells (Bregs), secreting of IL-10, have also been identified in the brain’s remote area following the stroke, where they contribute to neuroprotective processes and promote neurogenesis (Vadasz et al., 2013; Ortega et al., 2020). A new subset, age-associated CD11b^+^ B cells, is found to play a role in stroke recovery and influence microglial responses (Korf et al., 2022). However, some research highlighted the negative impact of B cells in post-stroke, such as auto-reactivity to myelin basic protein, cognitive delays, and ongoing inflammation. B cells were found to be related to post-stroke dementia (Doyle and Buckwalter, 2017), which was also demonstrated in a mouse model that the B cells may further directly impact cognition after stroke by penetrating into the infarct region and secreting IgA and IgG in the chronic phase after stroke (Doyle et al., 2015).

#### 3.2.2 Heterogeneity of B lymphocytes in IS

The peripheral immune response during the post-stroke stage has been found to correlate with cognitive changes, as identified through single-cell mass cytometry (Tsai et al., 2019). In flow cytometry, B cells can be selected using the cell markers CD3^−^CD19^+^CD20^+^. Key B cell subtypes relevant to stroke can be classified as follows: plasmablasts (CD27^+^CD38^+^), unswitched memory B cells (CD27^+^IgD^+^), switched memory B cells (CD27^+^IgD^−^), naïve B cells (CD27^−^IgD^+^), and double-negative B cells (CD27^−^IgD^−^) (Table 2). Although studies on peripheral B cells are rare, one study found a decrease in unswitched memory B cells in IS patients (Krishnan et al., 2021).

In scRNA-seq analyses, the marker genes for peripheral B cells are MS4A1 and CD79A. Aside from a slight increase in the expression of these genes in stroke, no additional significant findings have been reported regarding these markers (Cho et al., 2022). A mouse model of ischemic stroke revealed that B cells exhibit functional heterogeneity within the brain using scRNA-seq, with B1 and B2 cells distinguished using the markers *Ly6d*/*Cd79a* and *Ramp1*/*Lmo4*, respectively. Notably, the proportion of B1/B2 cells increased in mouse brains at 2 days and 14 days post-stroke compared to sham-operated controls (Garcia-Bonilla et al., 2024). However, the specific roles of these distinct subclusters require further investigation (Zheng et al., 2022).

### 3.3 NK cells

#### 3.3.1 Role of NK cells in IS

NK cells are a type of lymphocyte that mainly participate in the innate immune response, bridging the crosstalk between the central nervous system and peripheral immune cells (Zang et al., 2022). The rapid response of NK cells to ischemic insults may exacerbate brain infarction due to the increased cytotoxicity and inflammation (Crinier et al., 2020; Gan et al., 2014). In response to interferon-inducible protein-10 (IP-10), NK cells can also damage the BBB (Zhang et al., 2014). Besides, NK cells release granzymes and perforin upon direct contact with target cells, leading to cell death and exacerbating inflammation, which can worsen brain infarction following a stroke (Gan et al., 2014).

#### 3.3.2 Heterogeneity of NK cells in IS

Most clinical studies have demonstrated that the number of NK cells in IS changes dynamically. Typically, NK cells accumulate in the brain, while their numbers in peripheral blood can vary across different studies (Prinz and Priller, 2017). An increase in NK cells in peripheral blood is often observed, possibly due to the collection of samples during the acute phase post-stroke from patients who experienced relatively mild stroke symptoms (Cho et al., 2022; Yan et al., 2009). In contrast, patients with moderate to severe strokes did not show a significant change or experienced a decrease in circulating NK cell percentages following the stroke (Peterfalvi et al., 2009; Jiang et al., 2017).

In experimental models, neuroinflammation associated with ischemic brain injury exhibits variations during middle cerebral artery occlusion (MCAO), revealing distinct patterns of post-stroke immune cell behaviors (Zhou et al., 2013; Weil et al., 2021). It has been observed that stroke patients with higher numbers of circulating NK cells shortly after the event are more likely to develop infections, indicating that NK cell counts could serve as predictors for post-stroke infections (De Raedt et al., 2015). Furthermore, different mechanisms in the brain and periphery compromise NK cell functions after a stroke, with various modulators affecting NK cell numbers and responses, suggesting that targeting these specific pathways may provide new biomarkers for post-stroke infections (Liu et al., 2017).

In flow cytometry, the CD56^+^ CD16^−^ subset is typically considered the less mature of the two NK cell subsets and is commonly found in the lymph nodes, while the CD56^−^CD16^+^ subset is considered the more mature form, predominantly located in peripheral blood. These mature NK cells are highly cytotoxic and can directly kill target cells by releasing granules containing perforin and granzymes.

When combined with flow cytometry, DEGs can be identified through multi-omics approaches in IS. For example, miRNA-451a and miRNA-122-5p are significantly upregulated in circulating NK cells following ischemia stroke, as revealed by microRNA sequencing in human samples (Kong et al., 2020). In a mouse model, mass cytometry demonstrated that the p-P38 and p-STAT4 signaling pathways are increased in NK cells after stroke, while levels of p-pS6, p-EGFR, and p-STAT1 are decreased in these cells (Yao et al., 2021).

Typical marker genes for peripheral NK cells in stroke studies are *Gnly* and *Nkg7*. Compared to controls, a study identified the top 20 DEGs in the stroke dataset, with only two genes (*Cx3cr1* and *Gimap7*) upregulated in stroke patients (Cho et al., 2022) (Table 3). These DEGs suggest that NK cell activity was enhanced in stroke, as indicated by pathways such as NK cell signaling and PKR in interferon induction (Cho et al., 2022). The most prominent pathway identified was glucocorticoid receptor signaling, although no direct patterns among DEGs were observed. Analysis of the leading network among DEGs highlighted the upregulation of FAS ligand and downregulation of NFκB inhibitor alpha, further supporting the evidence of increased NK cell activity in stroke patients. Upregulated DEGs identified in the pathway analysis in stroke patients underscored activated NK cell signaling, involving multiple genes that contribute to this pathway (Table 4).

**Table 4:** Changes in differentiation trajectories of distinct cell types in mouse.

Cell types	Trajectories	Time points	Involved genes	Possible Involved function	References
Microglia	Mg1-2	Sham	*Siglech, P2ry12, Tmem119*	Homeostatic genes	Zheng et al. (2022), Li et al. (2022), Beuker et al. (2022), Garcia-Bonilla et al. (2024)
			*Jun, Fos, Erg1, Klf2, Klf4, Atf3*	Immediate early genes	
	Mg4-5	D02	*Spp1, Msr1, Lgals3*	Clearance of damaged cells	
			*Ccl2, Ccl12*	Chemokine genes	
	Mg3-6-7	D14	*Apoe, Cst7, Clec7a, Lyz2, Lgals3bp, Igf1, Lpl*	DAM genes	
			*Il1b, Nfkbiz, Cd83, Ccl4, Egr1, Fosb*	DIM genes	
	Mg4-5 & Mg3-6-7	Sham to D02 & Sham to D14	*Apoe, Lpl, Spp1, Clec7a, Cst7*	Microglial responses to demyelination	
Blood Monocytes and MdCs	Mo2	D02	*Saa3, Mmp8, Lcn2, Wfdc21, Lrg1, Chil3*	Transcriptional profile of ‘neutrophil-like’ Ly6C^hi^	Garcia-Bonilla et al. (2024)
	MdC1	D02	*Fabp5, Spp1, Gpnmb, Ctsl, Cd63, Ctsb, Ctsd, Arg1*	Stroke-associated macrophages genes	
	MdC2	D02	*Cxcl1, Cxcl2, Cxcl3*	Neutrophil chemoattractants genes	
			*Ptgs2, Il1b, Clec4e*	Pro-inflammatory genes	
	MdC6	D14	*Igf1*	Growth factor	
	MdC2,4,1-MdC6	D2 to D14	*Cxcl10*		
Blood neutrophils and brain granulocytes	Neu1, 2, 4	D02	*Vim, Cd14*		Garcia-Bonilla et al. (2024)
			*Mmp8, Retnlg, Lcn2, Ly6g*	Immature Neu signature	
			*Stfa1, Stfa2, Stfa3, Stfa2l1, BC100530*	stefinA family genes	
	Neu3	D14	*Ninj1, Cd300c2*	Neutrophil tissue infiltration	
			*Lst1, Creg1*	Cell growth inhibition	
			*Cd101*	Mature neutrophils	
	Gran1 – Neu1,2,4	Sham to D02	*Retnlg, Mmp8, Ly6g, Anxa1 and Lcn2*	Granulocytes in Sham brain and neutrophils in sham and D02	
	Gran2,3 – Neu3	D02 to D14	*Ccl3, Ccl4, Csf1 and Gadd45b*		

## 4 The application of scRNA-seq in stroke and post-stroke stages

### 4.1 The variation of differentiated trajectories during the acute and chronic phases of ischemic stroke in mice

**Figure 2:**
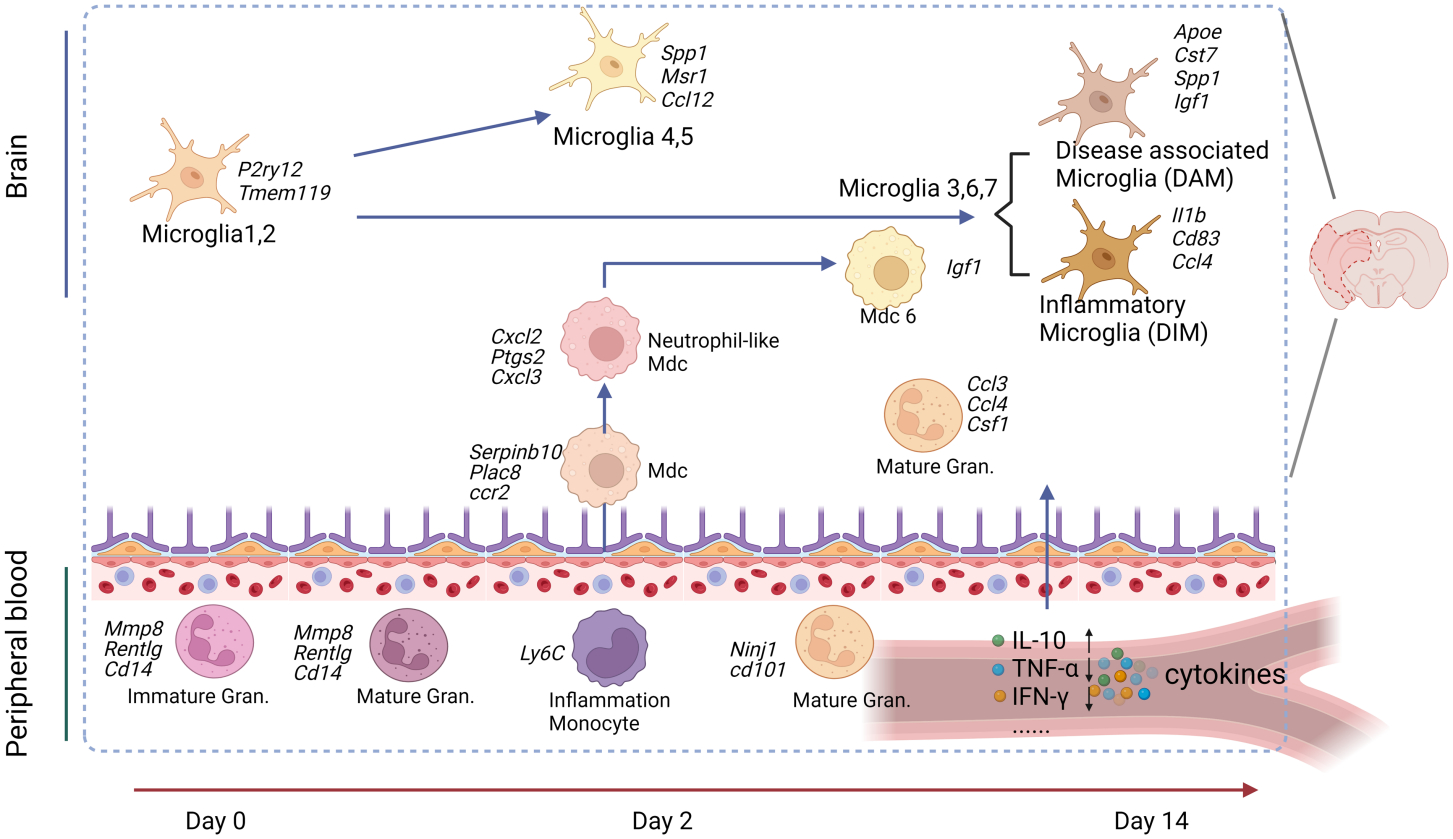
Peripheral immune cells connect to the brain cells after an experimental stroke. Peripheral blood granulocytes (Gran) at Day 0 and the acute phase (Day 2) were characterized by upregulation of immature neutrophil marker genes. Peripheral blood monocytes (Mo) were identified as patrolling or inflammatory Mo at all studied time points. Inflammatory Mo infiltrated the brain at the acute phase and differentiated in monocyte-derived cells (MdC), adopting several transcriptomic phenotypes from Day 2 to Day 14. Microglia (Mg) displayed specific transcriptomic signatures at each studied time point: Mg1,2 at baseline, proliferative and clearing Mg at the acute phase, and neuro-degenerative/disease-associated Mg (DAM) and disease inflammatory macrophage/microglia (DIM) at the subacute phase. Image created with Biorender.com, with permission.

The initial neuronal damage triggers a secondary inflammatory response, which exacerbates brain injury and neurological deficits. This response spans days to weeks, involving the activation of glial cells, border-associated macrophages (BAMs), and recruitment and infiltrating of the immune cells and endothelial cells (ECs). A recent study used scRNA-seq analysis of brain resident cells and peripheral leukocytes from mice at different time points (Day 2 and 14), illustrating their transcriptional diversity after IS (Garcia-Bonilla et al., 2024). This study identified numerous distinct cell clusters, including brain immune cells [microglia, BAMs, myeloid-derived cells (MdCs), and lymphoid cells]. The relative abundance of these cell clusters varied significantly with ischemia–reperfusion. Microglia cells are rapidly activated after IS, shifting to either a pro-inflammatory (M1) or anti-inflammatory (M2) state depending on the signals they receive (Cai et al., 2021; Wu et al., 2021). Simultaneously, circulating monocytes are recruited to the injury site, crossing the compromised BBB and infiltrating the infarcted tissue (Park et al., 2022). These monocytes differentiate into macrophage-like cells, further amplifying the local immune response. Microglia and macrophages may drive angiogenesis and oligodendrogenesis in the stroke brain through a paracrine mechanism (Jin et al., 2023). Given the critical role played by microglia cells, after IS, we mainly focused on the dynamics and diversity of microglia and MdCs in this review. These cells that were found with continuous pseudotime trajectories are summarized (Figure 2, Table 4).

#### 4.1.1 Microglial differentiation trajectories

Microglia re-clustering revealed eight distinct clusters with varying gene expression profiles over time. Early post-stroke (Day 2) microglia were characterized by genes involved in cell clearance and tissue repair, while later stages (Day 14) showed the upregulation of genes was associated with disease-associated microglia (DAM) and inflammatory responses (Garcia-Bonilla et al., 2024). Pseudotime trajectory analysis suggested distinct progression pathways for microglia were found from Sham to post-stroke conditions, including the transitional trajectories from the Mg1 cluster to either Mg2 (Sham), Mg4, and Mg5 clusters at Day 2 or Mg3, Mg6, and Mg7 clusters at Day 14 (Garcia-Bonilla et al., 2024). Involved genes and pathways are listed in Table 4, Figure 2.

#### 4.1.2 Differentiation of blood monocytes and monocyte-derived cells (MdCs)

MdCs showed a continuous differentiation trajectory from inflammatory monocytes to tissue macrophages with distinct transcriptional states, influenced by the local tissue environment rather than peripheral priming. A previous study showed the genes *Arg1, Clec4d,* and *Osm* had low expression in Ly6c^lo^ Mo and Ly6c^hi^ Mo_2 clusters, higher in the Mo/Mu_Spp1 cluster, and even greater in the Mo_Cxcl2 cluster, indicating dynamic changes in these genes followed the Mon/Mu states (Zheng et al., 2022). Trajectory analysis of peripheral blood and brain revealed a sequential transition of inflammatory peripheral blood Mo1 and Mo2 clusters into the brain MdC3 cluster, which was followed by transitions to MdC2, MdC4, and MdC1, ultimately differentiated into MdC6 (Garcia-Bonilla et al., 2024). These findings suggest that MdCs in the inflamed brain are derived from inflammatory monocytes. Both MdC1 and MdC6 appear to be tissue macrophages, while MdC2, MdC3, and MdC4 represent transitional phenotypes (Garcia-Bonilla et al., 2024) (Table 4).

#### 4.1.3 Blood neutrophil clusters and brain granulocyte clusters

The trajectory analysis of combined blood neutrophils and brain granulocytes revealed similarities. Gran1, the principal granulocyte cluster in Sham brain, closely resembled Neu1, Neu2, and Neu4, which were the dominant blood clusters in Sham and Day 2 samples (Garcia-Bonilla et al., 2024). In contrast, Gran2 and Gran3, the predominant blood granulocyte clusters observed on Day 14, strongly aligned with Neu3 (Garcia-Bonilla et al., 2024) (Table 4). Trajectory analysis suggested continuous recruitment from blood at early and late phases without in-tissue differentiation.

#### 4.1.4 Connections between peripheral blood cells and brain cells

Using non-scRNA-seq approaches, previous studies revealed some interactions between peripheral immune cells and brain cells. After cerebral ischemia, neutrophils do not infiltrate the brain parenchyma but remain confined to the luminal surfaces and perivascular spaces of cerebral blood vessels instead (Enzmann et al., 2013). In contrast, microglial proliferation within the CNS is facilitated by the recruitment of monocytes and macrophages originating from the meninges, choroid plexus, perivascular spaces, and bloodstream (Henning et al., 2009; Stroh et al., 2006). Studies using parabiotic mice demonstrated that the expansion of microglia post-stroke relies predominantly on the proliferation of CNS-resident cells (Li et al., 2013). Microglia and infiltrating monocytes or macrophages exhibit distinct roles in stroke recovery, particularly regarding their phagocytic activity and secretion of pro-inflammatory cytokines (Wicks et al., 2022; Kanazawa et al., 2017). γδT cells are drawn to the ischemic brain by IL-23-producing bone marrow-derived macrophages. These γδT cells secrete IL-17, further recruiting peripheral myeloid cells and exacerbating neuronal apoptosis in the penumbral region during the delayed phase of ischemia–reperfusion injury (Shichita et al., 2009). Interestingly, most γδT cells accumulate in the leptomeninges instead of penetrating the brain parenchyma, acting as regulators and controlling the migration of monocytes and neutrophils into the CNS (Ribeiro et al., 2019).

Currently, the application of scRNA-seq to simultaneously analyze peripheral blood and brain tissue in mouse stroke models is limited. Markedly, a recent study of IS using scRNA-seq covered both peripheral blood cells and brain cells (Garcia-Bonilla et al., 2024). This study elucidated that the transcriptomic profiles of brain myeloid cells, which originated from peripheral blood in the post-stroke period, remained significantly different from their origin, peripheral blood cells. In addition, another study on atherosclerosis, which may contribute to IS, revealed a distinct group of activated and differentiated T cells in plaques from symptomatic patients, which were characterized by T cells, including a distinct subset of CD4+ T cells (Fernandez et al., 2019). Some T cell subsets in these plaques presented markers of T cell exhaustion, while macrophages contained alternatively activated phenotypes, including subsets associated with plaque vulnerability. In plaques from asymptomatic patients, T cells and macrophages were activated and displayed evidence of interleukin-1β signaling (Fernandez et al., 2019).

### 4.2 Specific pathways and genes in peripheral blood and brain cells impacted by stroke

In the context of ischemic stroke, several key genes in brain cells exhibit significant changes. Microglial cells show upregulation of immediate early genes such as *Jun*, *Fos*, *Erg1*, *Klf2*, *Klf4*, and *Atf3*, which are involved in microglial surveillance functions (Table 5). Genes linked to microglial responses to demyelination, Alzheimer’s disease, and stroke, including *Apoe*, *Lpl*, *Spp1*, *Clec7a*, and *Cst7*, are also upregulated. Additionally, the expression of chemokine genes *Ccl2* and *Ccl12* increases. Genes related to cell proliferation (*Top2A*, *Mki67*, and *Stmn1*) and those affiliated with disease-associated microglia (DAM) such as *Apoe*, *Cst7*, *Clec7a*, *Lyz2*, *Lgals3bp*, *Igf1*, and *Lpl* are prominently expressed during the acute and subacute phases of stroke. Genes associated with inflammatory responses, like *Il1b*, *Nfkbiz*, *Cd83*, and *Ccl4*, are also significantly upregulated.

**Table 5:** Specific pathways and genes in brain cells that are impacted by stroke in mouse.

Cell types	Representative pathways and process	Representative DEGs	References
Microglia	Neutrophil chemotaxis/Apoptosis	Up: *Spp1, Lilrb4a, Cd72, Ccl7, Ccl12*	Zheng et al. (2022)
	Lysosome/Positive regulation of microglia cell migration	Down: *P2ry12, Siglech, Gpr34, Selplg, Hpgd*	
Astrocyte	Cellular responses to stress/Signal Transduction	Up: *Ccl4, Cdkn1a, Gfap, AY036118, Vim*	Zheng et al. (2022)
	Respiratory electron transport	Down: *Dbp, Gria2, Ntm, Itm2a, Appl2*	
Endothelial cell	Cellular response to chemical stimulus/Regulation of cell death	Up: *Ctla2a, Plat, Lrg1, Lcn2, Tmem252*	Zheng et al. (2022)
	Anion transport/Transport of small molecules	Down: *Rps27rt, Cxcl12, Spock2, Ifit3, Itm2a*	
Oligodendrocyte	Cytokine-mediated signaling pathway/Regulation of neuron apoptotic process	Up: *Serpina3n, Tma16, Gpd1, Phactr3, Klk6*	Zheng et al. (2022)
	Regulation of glial cell differentiation/ Negative regulation of neuron projection development	Down: *Hebp1,1700047M11Rik, Lpar1, Omg, Hs3st1*	
Ependymocyte	Response to steroid hormone/ Regulation of ion transport	Up: *Spp1, Ccl4, Ccl12, AY036118, Mt2*	Zheng et al. (2022)
	Oxidative phosphorylation/Ribonucleoprotein complex biogenesis	Down: *Defb11, Hist2h2aa1, Usp50, Ifi27l2a, Sostdc1*	
Pericyte	Regulation of secretion/Response to peptide	Up: *Ednrb, Il11, Timp1, Saa3, Ccl11*	Zheng et al. (2022)
	Transport of small molecules	Down: *Dbp, Itm2a, Cxcl12, Pltp, Tsc22d1*	
SMC	Cellular response to chemical stimulus/Cytokine-mediated signaling pathway	Up: *Ifitm1, Ccl4, Cdkn1a, Sdc4, Rasl11a*	Zheng et al. (2022)
	Regulation of calcium ion transmembrane transport/Relaxation of muscle	Down: *Fbxl22, Pln, Lbh, Crim1, Myh11*	
CAM	Toll-like receptor cascades/Chemokine receptor binding	Up: *Arg1, Saa3, Cxcl2, Msr1, Ccl6*	Zheng et al. (2022)
	Vasodilation /Amyloid-beta binding	Down: *Hist4h4, Hpgd, Maf, Lst1, Clec10a*	
Neutrophil	Neutrophil degranulation/IL-1 signaling pathway	Up: *Cxcl3, Ccrl2, Cxcl2, Marcksl1, Hcar2*	Zheng et al. (2022)
	regulation of peptidase activity	Down: *Ltf, Camp, Cd177, Ngp, Ly6g*	
MdC	Myeloid leukocyte migration/Acute-phase response	Up: *Arg1, Thbs1, Clec4n, Cxcl3, Ccl7*	Zheng et al. (2022)
	Cellular extravasation/Integrin-mediated signaling pathway	Down: *Rnase6, Ear2*	
DC	Cell chemotaxis/Positive regulation of defense response	Up: *Ifitm1, Ccr7, Fabp5, Pfkp, Ccl22*	Zheng et al. (2022)
		Down: *Rpl9-ps6, Clec4b1, Cd209a, Mgl2, Tnip3*	
Lymphocyte	Leukocyte apoptotic process/Metalloprotease DUBs	Up: *Ccl5, Dusp2, Ccl3, Gzma, Lck*	Zheng et al. (2022), Garcia-Bonilla et al. (2024)
	Cxcr3–Cxcl10 pathway/recruitment of CD8^+^ T cells	Down: *H2-Aa, Lyz2, Cd74, Ly6d, Xcl1*	
FB	Myoblast differentiation/Structural constituent of ribosome	Up: *Angptl4, Ccl4*	
SAMC	lipid metabolism and phagocytosis of myelin/phagocytosing and lipid-sensing	Up: *Spp1, Fabp5, Gpnmb, and Lpl*	Beuker et al. (2022)

SMC, smooth muscle cells; CAM, central nervous system (CNS)-associated macrophages; MdC, monocyte-derived cells; DC, dendritic cells; FB, fibroblast-like cells; SAMC, stroke-associated myeloid cell.

Although many studies have uncovered key pathways impacted by stroke, a vast majority of them were conducted in mice, while research on pathways in humans is limited. A study targeting the peripheral blood NK cell cluster found that among the top 20 differentially expressed genes (DEGs) in NK cells, two genes (CX3CR1 and GIMAP7) are upregulated, while 18 are downregulated, including genes such as CXCR4, DUSP2, and IL7R. The top five canonical pathways identified from the DEGs include NK cell signaling, PKR in interferon induction, and p38 MAPK signaling, all suggesting enhanced NK cell activity during stroke (Cho et al., 2022). For monocytes, the overall trend towards the inactivity of monocytes in stroke patients is linked to the strongly inhibited EIF2 signaling pathway. One subcluster of monocytes, characterized by specific DEGs relevant to neurological disorders, could potentially serve as prognostic or diagnostic biomarkers for ischemic stroke or its treatment (Cho et al., 2022).

In mouse models, studies found that 275 differentially expressed genes (DEGs) with significant changes in gene expression in microglia were identified, suggesting their dominant role in post-stroke neuroinflammation (Zheng et al., 2022). Key pathways identified include the neutrophil chemotaxis and apoptosis signaling pathway in microglia, signal transduction in astrocytes, regulation of neuron apoptotic processes in oligodendrocytes, and transport of anion and small molecules in endothelial cells (Shi et al., 2019). Additionally, the upregulation of chemokines and cytokines like *Ccl7* and *Ccl12* in microglia, *Ccl4* and *Cdkn1a* in astrocytes, and *Ccl4* in ependymal cells underscores the molecular mechanisms driving inflammation post-stroke (Zheng et al., 2022). By contrast, microglial homeostatic genes (*P2ry12, Tmem119*) were downregulated after ischemia stroke (Zheng et al., 2022; Beuker et al., 2022) (Table 5).

Currently, scRNA-seq is mostly conducted in mouse models for stroke-related studies, with relatively few studies directly focusing on human stroke patients. A recent study discussed the transcriptional process in peripheral blood leukocytes, showing varying degrees of conservation between mice and humans, particularly at different phases of the stroke response (Garcia-Bonilla et al., 2024; Mestas and Hughes, 2004). During the acute phase, only a small fraction (9%) of pathways were shared between human and mouse datasets, indicating significant differences in early immune responses. However, in the subacute phase, there was a higher overlap (47%) of pathways, suggesting a more conserved immune response between species as the stroke progresses (Garcia-Bonilla et al., 2024). Key pathways commonly enriched in both humans and mice include those related to adaptive immunity, such as antigen processing and presentation, TH17 cell differentiation, and B cell receptor signaling, as well as pathways involved in phagocytic function, protein degradation, cytokine and inflammatory signaling, apoptosis, and oxidative phosphorylation (Garcia-Bonilla et al., 2024). The above pathways are critical in recruiting and regulating immune cells during inflammation.

### 4.3 scRNA-seq enables exploration of the potential pathological mechanism and discovery of personalized treatment strategies for stroke

#### 4.3.1 Gene and pathway for finding the target

scRNA-seq can identify differentially expressed genes and pathways in specific cell types during different stages of stroke. This helps pinpoint critical molecular players in the disease process, such as pro-inflammatory cytokines, chemokines, and signaling pathways. Specifically, these signaling pathways are involved in potential pathological mechanisms, such as cell death and survival. Identifying specific markers and pathways unique to harmful cell states (e.g., pro-inflammatory microglia or infiltrating neutrophils) enables the development of targeted therapies, which can aim to inhibit the detrimental effects while preserving or enhancing protective mechanisms. For instance, the IFN-p38 MAPK-Nuclear factor kappa B (NFκB) pathway was the primary signaling cascade of activated NK cells, which could be suggested as a therapeutic target of ischaemic stroke (Cho et al., 2022). Moreover, insights into pathways and genes involved in tissue repair and neurogenesis can inform the development of regenerative strategies. Promoting the differentiation of neural progenitor cells or modulating supportive glial cell functions to enhance recovery. For example, extracellular vesicle-mediated *Lgals9* delivery improved the long-term functional recovery in photothrombotic stroke mice, suggesting *Lgals9* could be a potential treatment target (Han et al., 2024). Understanding how immune cells, neurons, and glial cells communicate during and after a stroke can reveal targets for modulating these interactions to reduce damage or promote recovery.

#### 4.3.2 The dual role of immune cells to aid treatment

The dual roles of immune cells in both damaging and repairing tissues have been found in stroke and post-stroke comorbidities. By tracing the differentiated trajectories of cells, scRNA-seq shows how different cell types evolve post-stroke over time. For example, microglia are found with the transition states from homeostatic to reactive, which can help us understand the progression of inflammation and tissue repair mechanisms (Garcia-Bonilla et al., 2024). By defining distinct transcriptional profiles associated with different immune cell functions, scRNA-seq can help identify distinct cell subgroups that specifically respond to IS, further revealing key genes, pathways, or regulators in the specific subgroup of immune cells. For example, a study of human heart failure samples uncovered significant macrophage heterogeneity by scRNA-seq, including a highly conserved lipid-associated macrophage subtype that plays a dual role in different pathological conditions, with both beneficial and detrimental effects (Jiang et al., 2024). Another study found the dual role of TREM2 in microglial cells in Alzheimer’s disease, where TERM2 was used to be considered to play only protective roles for Alzheimer’s disease (Rachmian et al., 2024). By pinpointing the dual roles by scRNA-seq, mouse models could be subsequently implemented to confirm the functions of highlighted genes, pathways, or regulators, ultimately serving as potential therapeutic targets to modulate immune responses in stroke.

Understanding the dual role of immune cells in both damaging and repairing can help design immunomodulatory therapies. These therapies prospectively reduce the initial harmful inflammatory response while supporting the beneficial aspects of immune activity in later stages. Furthermore, scRNA-seq allows the profiling of individual patient responses to stroke, which can lead to personalized treatment approaches based on the specific cellular and molecular landscape of stroke from individual patients, improving the efficacy of interventions.

### 4.4 Comparison of RNA sequencing technologies in stroke-related studies

Different RNA sequencing methods offer distinct advantages and/or limitations in IS-related studies. Bulk RNA sequencing (bulk RNA-seq) provides a comprehensive overview of gene expression across a large population of cells, enabling the identification of broad transcriptional changes. In particular, purified or sorted bulk RNA-seq approaches can enrich specific cell groups before sequencing. Purified bulk RNA-seq, based on tissue or organ homogenates, results in RNA extracted from a more heterogeneous mixture of cells (Table 6). This method can obtain a high yield of RNA from larger, mixed-cell populations, making it useful for analyzing tissue-wide transcriptomic profiles. However, the lack of specificity in isolating distinct cell types limits the resolution and could omit gene expression patterns from specific cell types. Sorted bulk RNA-seq involves the physical separation of distinct cell populations, typically using techniques like fluorescence-activated cell sorting (FACS) or magnetic-activated cell sorting (MACS). This approach ensures that only specific cell types are defined by surface markers with increased resolution, allowing for the identification of cell-type-specific transcriptomic signatures. However, RNA yield from sorted populations may be lower, especially when targeting rare cell types, which can impact downstream data quality. Moreover, the sorting process may stress or damage cells, potentially altering their transcriptional profiles.

**Table 6:** Comparison of RNA sequencing technology in stroke-related studies.

Technology	Advantages	Limitations
Bulk-RNA sequencing	1. Comprehensive overview of gene expression	1. Loss of cellular resolution
	2. Cost-effectiveness	2. Limited to average expression levels
	3. Higher sensitivity for lowly expressed genes	3. Lower precision for purified bulk RNA-seq
	4. Faster and less expensive for purified bulk RNA-seq (e.g., density centrifugation or magnetic beads)	4. Slower and more expensive for sorted bulk RNA-seq due to require sorting marker selection and specialized equipment (e.g., flow cytometers)
	5. high specificity and accuracy of rare subpopulations for sorted bulk RNA-seq	
Single-cell RNA sequencing (scRNA seq)	1. The entire transcriptome, both nuclear and cytoplasmic mRNA	1. Requires fresh, viable cells
	2. Higher sensitivity to gene expression	2. Tissue Dissociation Challenges (e.g., brain samples)
	3. Better capture the dynamic states of cells	3. Transcriptomes perturbed during isolation
	4. A broader range of RNA species	4. No spatial information preserved
Single-nucleus RNA sequencing (snRNAseq)	1. Flash-frozen or fixed tissue can be used	1. Incomplete transcripts measured
	2. Applicable to difficult dissociate tissue (e.g., brain samples)	2.Reduced sensitivity in detecting low-abundance transcripts
	3. Minimization of dissociation artifacts	
	4.Less bias against fragile cells	
Spatial RNA sequencing	1. Knowing spatial relationships and microenvironments of cellular	1. Limited transcript coverage
	2. Retains information about tissue architecture	2. Higher cost and not scalability
	3. Identification of cell niches	

Bulk RNA-seq cannot distinguish different cell types, which may obscure critical cellular heterogeneity and the contributions of rare cell populations. In this case scenario, scRNA-seq has a high resolution of gene expression at the single-cell level, identifying distinct cell types and rare subpopulations that may play key roles in stroke pathology. The major limitation of scRNA-seq is the requirement for fresh, viable cells, which could be challenging when working with tissues that are difficult to dissociate, such as the brain. The advantages and disadvantages of these RNA sequencing methods, as well as variable marker genes or treatment targets for IS found in different studies, are summarized in Tables 1, 6.

The scRNA-seq analysis relies solely on RNA expression to infer cell identity and function (Kharchenko, 2021). This can result in an incomplete representation of the cellular phenotype, especially when mRNA expression levels do not directly correlate with protein abundance. Moreover, while scRNA-seq provides a detailed snapshot of mRNA levels within individual cells, it overlooks post-transcriptional modifications, translational control, and protein stability, all of which play critical roles in determining the actual functional state of the cell (Zhao et al., 2021).

To address these issues, CITE-seq (cellular indexing of transcriptomes and epitopes by sequencing) was developed, representing a hybrid advancement in single-cell sequencing technology that integrates transcriptomic profiling with protein expression analysis (Stoeckius et al., 2017). Positioned within the realm of multimodal single-cell approaches, CITE-seq expands upon traditional scRNA-seq by simultaneously measuring gene expression and surface protein markers using oligonucleotide-tagged antibodies (Stoeckius et al., 2017). This dual modality enables a more comprehensive cellular characterization, effectively addressing several limitations of conventional single-cell RNA sequencing.

CITE-seq allows for the identification of specific proteins that may not be detectable at the transcript level by scRNA-seq, either due to their absence or extremely low expression levels (e.g., CD16). This limitation in traditional scRNA-seq often makes it difficult to detect such proteins. However, CITE-seq can not only detect these proteins through antibody-based barcoding but also allows for cross-validation of results with flow cytometry, thereby providing a more comprehensive and accurate profile of cellular phenotypes (Fernandez et al., 2019).

For instance, CITE-seq combined with flow cytometry enriched the CD45+ cells in plaque tissue and blood, further elucidating key differences in immune molecular mechanisms and signaling pathways (Fernandez et al., 2019). This study found that plaques from symptomatic patients were characterized by a distinct subset of activated and differentiated CD4+ T cells.

Single-nucleus RNA sequencing (snRNA-seq) allows the analysis of nuclei from frozen or preserved tissues, making it particularly useful for studying tissues that are difficult to dissociate. The application of snRNA-seq could overcome some of the challenges associated with scRNA-seq, but it mainly captures the nuclear transcriptome, which may not fully represent the cytoplasmic mRNA content, potentially leading to an incomplete picture of gene expression.

Spatial RNA sequencing (spatial RNA-seq) offers another dimension by providing spatial context to gene expression data, allowing researchers to correlate gene expression with tissue architecture. This technique is particularly valuable in understanding the spatial organization of cell types and their interactions within the ischemic brain. However, spatial RNA-seq may have lower resolution than single-cell techniques, and it is generally more costly and technically demanding. Each method presents a unique set of trade-offs, and the choice of technique should be guided by the specific research question and the nature of the tissue being studied. For instance, spatial transcriptomics combined with scRNA-seq allows the mapping of immune cells’ spatial distribution in the brain post-stroke. In a study of analysis on the ischemic hemisphere in mice after stroke, cell types identified by scRNA-seq confirmed and enriched the spatial annotation of ischemia-associated gene expression in the peri-infarct area of the ischemic hemisphere (Han et al., 2024). This combined technology allows us to visualize the tissue’s transcriptional landscape and identify gene expression profiles linked to specific histologic entities.

## 5 Perspectives

In this review, we summarized the roles and heterogeneity of PBMCs and biomarkers used in flow cytometry and scRNA-seq technology. Specifically, we underscored the dual roles, changes in differentiation trajectories, and specific pathways and genes of distinct cell types impacted by stroke. To offer potential research approaches and strategies for ischemic stroke by scRNA-seq, we also summarized the application of scRNA-seq, including the connections between PBMCs and brain cells during and after stroke, highlighting the high-resolution view of cellular heterogeneity and dynamics in ischemic stroke. By identifying specific cell types, pathways, and gene expressions involved in the disease process, scRNA-seq provides valuable insights that could inform the development of targeted therapies and improve stroke outcomes (Figure 1).

From future perspectives, scRNA-seq can guide the development of new drugs by identifying key pathways and genes involved in the immune response and tissue repair post-stroke (Van de Sande et al., 2023). For example, targeted genes involved in microglial activation or endothelial cell repair could yield novel therapeutic agents (Garcia-Bonilla et al., 2024). The CXCL10-CXCR3 signaling pathway could also be a target involved in the recruitment of immune cells to the ischemic brain, helping reduce neuroinflammation and limit secondary brain damage (Zheng et al., 2022). Interleukins such as IL-1β, TNF-*α*, and IL-6 can help mitigate the inflammatory response and improve outcomes in stroke patients, which could be another immunomodulation target (Deng et al., 2024).

Notably, the change in peripheral blood cell counts at the post-stroke stage may vary across different studies. For example, NK cells were found to be reduced in stroke patients in a study (Peterfalvi et al., 2009; Jiang et al., 2017), but it was found to be an increasing trend in another study (Cho et al., 2022). This observation could be linked to the mild symptoms of patients, but it may also occur due to the study conducted at different stages of a cell type. Moreover, investigations using different technologies (e.g., flow cytometry vs. scRNA-seq) may result in different abundances of the same cell type, possibly due to the different markers used in the measurement.

As the scRNA-seq technology is still developing, limitations are inevitable in the application. Due to influences such as RNA expression, post-translational modifications, cell surface trafficking, protein stability, and proteolytic changes, the correlation between mRNA and surface protein expression is relatively weak in immune cells (typically around 40%) (Schwanhäusser et al., 2011). Imperfections in mRNA capture and prevalent mRNA drop-outs further diminish the reliability of linking gene expression directly with surface protein levels (Baysoy et al., 2023). Despite the precise cell typing provided by flow cytometry for PBMCs, scRNA-seq often struggles to distinguish between major immune cell types like CD4^+^ and CD8^+^ T cells and NK cells (Zernecke et al., 2020; Mogilenko et al., 2021). A few approaches were implemented to mitigate the above limitations, such as snRNA-seq, flow cytometry combined with scRNA-seq, CITE-seq, and so on. Specifically, after DEGs/pathways were identified by scRNA-seq as potential biomarkers, flow cytometry can complement protein level validation to assess whether the identified biomarkers are indeed translated into proteins and to quantify their levels in a larger number of cells (Fernandez et al., 2019). Moreover, flow cytometry can also be paired with functional assays, such as cytokine secretion or cell proliferation assays, to assess the biological significance of the identified biomarkers. After describing the transcriptional landscape of PBMC in post-stroke disease by scRNA-seq, future experiments such as mechanistic examination can be conducted to investigate the pathological process during the stroke to identify new potential therapeutic targets.

In addition, translating scRNA-seq findings into clinical practice is not straightforward, where robust validation of biomarkers and therapeutic targets in larger patient cohorts is needed before they can be used in clinical settings. In this context, integrating AI technologies with scRNA-seq is promising for reducing the sample size and long-term validation before clinical trials and personalized treatment. Using AI technologies, such as deep learning on scRNA-seq data, automated data analysis could capture key information that could be potentially overlooked by manual analysis, substantially improve the predictive accuracy, facilitate real-time clinical decision-making, and bridge the gap between high-dimensional single-cell data and actionable clinical insights (Yang et al., 2022). This approach not only accelerates the translation of research findings into clinical practice but also enhances the precision and effectiveness of treatments. In summary, the application of single-cell sequencing to investigate peripheral blood cells after an ischemic stroke offers unparalleled insights into the dynamic roles of immune cells, enabling the identification of specific roles of different cell types with differentiation trajectories. This enhanced understanding is critical for developing biomarker that could predict the peripheral immune response to stroke inflammatory and recovery, developing new drugs and clinical trials on stroke treatments.
